# HEALTH INSURANCE LITERACY, HEALTH STATUS, AND CONCERNS ABOUT AFFORDABILITY OF HEALTH INSURANCE NEAR RETIREMENT

**DOI:** 10.1093/geroni/igz038.042

**Published:** 2019-11-08

**Authors:** Aaron M Scherer, Erica Solway, Preeti Malani, Jamie Luster, Jeffrey Kullgren, Matthias A Kirch, Dianne Singer, Renuka Tipirneni

**Affiliations:** 1 University of Iowa Carver College of Medicine, Iowa City, Iowa, United States; 2 Institute for Healthcare Policy and Innovation, University of Michigan, Ann Arbor, Michigan, United States; 3 University of Michigan Medical School, Ann Arbor, Michigan, United States; 4 Child Health Evaluation and Research Center, University of MIchigan, Ann Arbor, Michigan, United States

## Abstract

Results from the NPHA highlight the link between health insurance affordability concerns and delaying/forgoing health care near retirement. We sought to determine factors associated with health insurance affordability concerns for US adults age 50-64. We regressed little/no confidence in health insurance affordability in retirement/within the next year on health insurance literacy, age, gender, race/ethnicity, income, employment, education, and health status. Factors associated with greater health insurance affordability concerns included lower health insurance literacy—measured via confidence knowing health insurance terms (aOR=1.78, p=0.035), identifying covered services (aOR=1.81, p=0.038), and finding out service costs (aOR=2.69, p<0.001)—female gender (aOR=1.73, p=0.001), and fair/poor health (aOR=1.88, p=0.020). Factors associated with fewer health insurance affordability concerns included African-American race (aOR=0.55, p=0.038) and higher income (aOR=0.34, p<0.001). These results suggest that it may be possible to reduce health insurance affordability concerns and delayed/forgone care by improving adults’ confidence in understanding and using health insurance.

